# Supplementary material for: Profiling the regulatory landscape of sialylation through miRNA targeting of CMP- sialic acid synthetase
Source: J Biol Chem. 2025 Feb 24;301(4):108340. doi: 10.1016/j.jbc.2025.108340 (PMC11982980; doi:10.1016/j.jbc.2025.108340)
Supplement: Supporting information [file mmc1.docx]

Supporting information on main text, figures, and methods

**Title**: Profiling the Regulatory Landscape of Sialylation through miRNA Targeting of CMP- Sialic Acid Synthetase

**Authors:** Faezeh Jame-Chenarboo^1^, Joseph N. Reyes^1^, Thusini Uggalla Arachchige^1^, Lara K. Mahal^1^*

**Affiliations:** ^1^Department of Chemistry, University of Alberta, Edmonton, Alberta, Canada T6G 2G2

* **Corresponding Author:** lkmahal@ualberta.ca

This file contains:

Figs. S1-S8

Tables S1-S2

Figure S1. pFmiR-CMAS map and sequence. (A) pFmiR-CMAS plasmid map. (B) CMAS

**A**

**B**

5’-AAATTAGCGTAATATTGAGAAAAAAATGATACAGCCTTCTTCAGCCAGTTTGCTTTTATTTTTGAT

TAAGTAAATTCCATGTTGTAATGTTACAGAGAGTGTGATTTGGTTTGTGATATATATATATTGTGCTCT

ACTTTTCTCTTTACGCAAGATAATTATTTAGAGACTGATTACAGTCTTTCTCAGATTTTTAGTAAATGC

AAGTAAGAACATCATCAAAGTTCACTTTGTATTGTACCCTGTAAAACTGTGTGTTTGTGTGCTTTCAAA

GATGTTGGGATTTTATTTATCTGGGGACAGTGTGTATGGTAAGACATGCCCTTCTATTAATAAAACT

ACATTTCTCAAACTTGA-3’

3'UTR sequence. Sequence contains 357 base pairs.

Figure S2. Transcript expression level of CMAS in different tissues. Transcript expression map is from GTex portal (<https://www.gtexportal.org/home/transcriptPage> for CMAS, downloaded 10/2024).

Figure S3. miRFluR assay hits for CMAS. (A) Bar graph of miRNA hits from 95% confidence interval for CMAS. Data are normalized over median. Error bars represent standard deviation of technical replicates (n=3).

**Figure S4. Validation of miR-625-5p as new NTC.** (A) miRNA mimics of median controls (miRs: -625-5p (NTC), -548ay-3p -indicated on the blot) were transfected into MDA-MB-231 cells (50 nM miR, 48 h) prior to Western blot analysis. Representative data is shown. (B) Bar graph shows quantification of duplicate Western blot results as obtained in A. Signals were normalized to total protein on Ponceau. (C) Ponceau of Western blot shown in A. (D) Complete blot of Western shown in A, median control miRNAs are indicated. One-sample *t*-test was used to compare Ponceau normalized data for miRNAs to NTC, NS: not significant, *p*-values are indicated on the graph.

**Figure S5.** **Ponceau and whole Western blots for data shown in Fig. 3A and 3C.** (A, C) Ponceau staining of blots used in Fig. 3A, 3C (A: MDA-MB-231; C: MDA-MB-436). (B, D) Whole Western blot for data shown in Fig. 3A, 3C (B: MDA-MB-231; D: MDA-MB-436). (E, F) anti-CMAS antibody validation. (E) Ponceau staining of blot shown in F in MDA-MB-231. (F) Whole Western blot of siRNA validation of CMAS antibody. (G) miRNA mimic of median control (miR-625-5p: NTC) and mock miR transfection control (Mock) were transfected into MDA-MB-231 cells (50 nM miR, 48 h) prior to Western blot analysis. Representative data is shown. (H) Bar graph shows quantification of duplicate Western blot results as obtained in G. Signals were normalized to total protein on Ponceau. (I) Ponceau of Western blot shown in G. (J) Complete blot of Western shown in G, median control miRNA and mock are indicated. One-sample *t*-test was used to compare Ponceau normalized data for miRNAs to NTC, NS: not significant, *p*-values are indicated on the graph. *Note, data in 3A and 3C correspond to triplicate experiments, but as the alternate median control (miR-548ay-3p) and lysate experiments were only done in duplicate, graphs here reflect duplicate data.*

**Figure S6**. **Ponceau and Western corresponding to Fig. 4B in MDA-MB-231 cells**. (A) Ponceau staining of blot used in Fig. 4B. (B) Whole Western blot for data shown in Fig. 4B.

**Figure S7.** **Ponceau and Western corresponding to Fig. 6A and 6D in SU.86.86 cells**. (A, C) Ponceau staining of blots used in Fig. 6A, 6D (A: CMAS; C: ST6GAL1). (B, D) Whole Western blot for data shown in Fig. 6A, 6D (B: CMAS; D: ST6GAL1). (E) miRNA mimics of median controls (miRs: -625-5p (NTC), -548ay-3p -indicated on the blot), mock miR transfection control (Mock) and lysate were transfected into SU.86.86 cells (50 nM miR, 48 h) prior to Western blot analysis. Representative data of duplicates is shown. (F) Bar graph shows quantification of duplicate Western blot results as obtained in E. Signals were normalized to total protein on Ponceau. (G) Ponceau of Western blot shown in E. (H) Complete blot of Western shown in E, median control miRNA and mock are indicated. One-sample *t*-test was used to compare Ponceau normalized data for miRNAs to NTC, NS: not significant, *p*-values are indicated on the graph. *Note, data in 6A and 6D correspond to triplicate experiments, but as the alternate median control (miR-548ay-3p) and lysate experiments were only done in duplicate, graphs here reflect duplicate data.*

**Figure S8.** **Validation of antibody for CMAS in SU.86.86 cells.** (A) Western blot analysis of SU.86.86 cells treated with non-targeting pooled (NTP) or pooled siRNA targeting CMAS (si-CMAS). Cells were transfected into with varying amounts of siRNA (25, 50 nM) for 48 h prior to Western blot analysis. (B) Bar graph shows quantification of results shown in A. Signals were normalized to total protein on Ponceau. (C) Ponceau of Western blot shown in A. (D) Complete blot of Western shown in A.

Table S1. Statistical significance for Western blot experiments using both one-sample *t*-test and paired Student *t*-test.

| miRNA/ anti-miR | one-sample *t*-test | paired *t*-test | cell line |
| --- | --- | --- | --- |
| Fig. 3B | | | |
| up-miR | | | MDA-MB-231 |
| 196a-3p | 0.1 (NS) | 0.2 (NS) |  |
| 941 | 0.02 | 0.1 (NS) |  |
| 301b-3p | 0.0004 | 0.03 |  |
| 31-3p | 0.01 | 0.0003 |  |
| down-miR | | |  |
| 200c-5p | 0.003 | 0.007 |  |
| 323a-3p | 0.03 | 0.04 |  |
| 550a-3p | 0.002 | 0.005 |  |
| Fig. 3D | | | |
| up-miR | | | MDA-MB-436 |
| 196a-3p | 0.01 | 0.08 (NS) |  |
| 941 | 0.01 | 0.03 |  |
| 301b-3p | 0.0005 | 0.04 |  |
| 31-3p | 0.01 | 0.03 |  |
| down-miR | | |  |
| 200c-5p | 0.004 | 0.009 |  |
| 323a-3p | 0.01 | 0.04 |  |
| 550a-3p | 0.02 | 0.05 |  |
| Fig. 4C | | | |
| anti-up-miR | | | MDA-MB-231 |
| anti-301b-3p | 0.009 | 0.04 |  |
| anti-31-3p | 0.02 | 0.05 |  |
| anti-941 | 0.003 | 0.03 |  |
| Fig. 6B | | | |
| up-miR | | | SU.86.86 |
| 196a-3p | 0.004 | 0.02 |  |
| 31-3p | 0.0007 | 0.02 |  |
| down-miR | | |  |
| 200c-5p | 0.02 | 0.003 |  |

**Table S2.** Primer sequences for PCR amplification of wild-type (WT) 3'UTR (A) and for site directed mutagenesis of CMAS (B).

| Primer Name | Sequence (5' → 3') | Sample |
| --- | --- | --- |
| (A). PCR amplification of CMAS 3'UTR | | |
| CMAS-FWD^a^ | AGTAAATGCAAGTAAGAACATCATCAAAG | gDNA, HEK-293T |
| CMAS-REV^a^ | CCCCAGATAAATAAAATCCCAACAT | gDNA, HEK-293T |
| (B). PCR amplification of CMAS mutant 3'UTRs | | |
| 200c-5p-CMAS-MUT-FWD | atgcattctgtTGCCCTTCTATTAATAAAAC | pFmiR-CMAS |
| 200c-5p-CMAS-MUT-REV | acacactgtcccCAGATAAATAAAATCCCAACATC | pFmiR-CMAS |
| 550a-3p-CMAS-MUT-FWD | atgcattctgtTGCCCTTCTATTAATAAAAC | pFmiR-CMAS |
| 550a-3p-CMAS-MUT-REV | acacactgtcccCAGATAAATAAAATCCCAACATC | pFmiR-CMAS |
| 31-3p-CMAS-MUT-FWD | aaccattcagttGCCCTTCTATTAATAAAACTAC | pFmiR-CMAS |
| 31-3p-CMAS-MUT-REV | tgtgtgactggcCAGATAAATAAAATCCCAACATC | pFmiR-CMAS |
| 301b-3p-CMAS-MUT-FWD | ataaataacacgacTACTTTTCTCTTTACGCAAG | pFmiR-CMAS |
| 301b-3p-CMAS-MUT-REV | aataacagtttcgaAATCACACTCTCTGTAAC | pFmiR-CMAS |

[a] FWD, forward; REV, reverse.

**Dataset S1. miRFluR data for pFmiR-CMAS**. Tab 1. Total pre-QC dataset. Tab 2. Total post-QC dataset. Tab 3. Log_10_ transformed data and Z-scores. Tab 4. Final hitlist. Tab 5. Comparison of all NTC1 data within library.

**Dataset S2. miEAA Analysis**. Tab 1. Pathway analysis. Tab 2. Disease analysis.
